# Supplementary material for: Quality-adjusted life year comparison at medium term follow-up of endovascular versus open surgical repair for abdominal aortic aneurysm in young patients
Source: PLoS One. 2021 Dec 2;16(12):e0260690. doi: 10.1371/journal.pone.0260690 (PMC8639078; doi:10.1371/journal.pone.0260690)
Supplement: Attachment — Submitted filename: Response to Reviewers.docx [file pone.0260690.s003.docx]

Robert Jeenchen Chen, MD, MPH

Academic Editor

*PLOS ONE*

10/26/2021

Dear Dr. Chen:

On behalf of the authors, I would like to thank you and the reviewers for the valuable and helpful comments on our submitted manuscript, “[**Quality Adjusted Life Year comparison at medium term follow-up of endovascular versus open surgical repair for abdominal aortic aneurysm in young patients**]” (PONE-D-21-24097). The title of the paper has been changed from [Comparison of the quality of life and cost-effectiveness of endovascular versus open surgical repair for abdominal aortic aneurysm in young patients] to the above.

We have carefully reviewed the comments and have revised the manuscript accordingly. Our responses are provided in a point-by-point manner, and changes to the manuscript are shown in red, which are also visible through the Track Changes function.

We would be happy to make further corrections if necessary, and look forward to hearing from you soon.

Sincerely,

Tae Won Kwon, MD, PhD

Division of Vascular Surgery, Department of Surgery

Asan Medical Center

University of Ulsan College of Medicine

88, Olympic-ro 43-gil

Songpa-gu, Seoul 05505, Republic of Korea

Phone: +82-2-3010-3492

E-mail: [twkwon2@amc.seoul.kr](mailto:twkwon2@amc.seoul.kr)

Response to Editor

Thank you for your inviting to submit a revised version of the manuscript. We submitted the separate files with an appropriated title. We adjusted the style of the manuscript to the style requirements of PLOS ONE and updated the ORCID ID for the corresponding author.

**Response to Reviewer #1**

Thank you for your review of our paper. We have answered each of your points below.

Comment 1:

First of all, congratulate the authors for the idea and the work presented. There are some controversial aspects about the advantages of one technique or another in certain groups of patients and the results of the treatment in a short, medium and long term.
Next, a series of recommendations are made to the authors for their consideration.
Why did they choose that period of time for patient inclusion and on which they based the period of time necessary for patient follow-up?

Response:

Stent-graft was first introduced in the early 1990's. Since then, EVAR's popularity soared after 2007 and our center utilized this method during 2012. This is also the reason why our centre started to recruiting around this time. Moreover, when this study began in 2019, authors wanted to see a 3-year mid-term result from 2016, which is why the study chose these particular years. Survival rate for DREAM trial and EVAR 1 trial were 3 and 4 years, respectively. This is the reason why authors deemed that waiting for 3 years made logical sense.

Comment 2:
Line 165 statistical analysis It is important that the authors indicate how the sample size was calculated or, failing that, with the analyzed sample, how we can be sure that the data is statistically significant and relevant?

Response:

Our current findings were obtained at a single center, resulting in a small sample size, which limits the overall generalizability of our results. As a retrospective study, however all patients who met the predefined inclusion criteria were included.

Comment 3:
Authors should explain what the operator's experience was, and what it depended on being performed by a radiologist or vascular surgeon.

Response:

There were two vascular surgeons and both had 20 plus years of experiencing performing OSR. There is no difference in following a single protocol from the same hospital setting. As for EVAR, one surgeon and two radiologists performed the evaluation. They have often operated together and discussed all results together, which makes next to zero deviation in technique. We have added some description of this method to the method section. (page 5, lines 120 – 123)

*“All OSRs were performed by two vascular surgeons. There is no difference in following a single protocol from the same hospital setting. EVARs were performed by one vascular surgeon or two radiologists. They have often operated together and discussed all results together.”*

Comment 4:
line 67 please include the reference.

Response:

We have added the reference. Reference was made to the 2020 NICE guidance.

Comment 5:
Please define what you understand by high anesthetic risk and how you estimated the risk of each patient.

Response:

In the case of comorbid diseases, the possibility of general anesthesia was evaluated by collecting the opinions of experts in each department, and patients evaluated as high-risk groups or contraindications during general anesthesia were excluded. We have added some description to the method section. (page 5, lines 101 – 104)

*“The possibility of general anesthesia was evaluated by collecting the opinions of experts in each department, and patients evaluated as high-risk groups during GA were excluded. Even when EVAR was always performed under LA, we prepared for GA, to be possible just in case.”*

Comment 6:
It would be important for the authors to include the cause of the aneurysm (aetiology), that is, if it is of atherosclerotic origin related to hypertension or is it due to an arteriopathy.

Response:

Only patients with degenerative AAA were included. Cases where emergency surgery such as traumatic and infectious AAA were excluded. Cases where EVAR were not possible such as inflammatory AAA were also excluded. These descriptions were added to the method. (page 3, lines 95 – 97)

*“2) patients requiring emergency surgery due to rupture or infection and requiring only OSR owing to the inflammation (only degenerative AAA were included)”*

Comment 7:
Were any criteria used for the EVAR to be performed by radiologists or vascular surgeons?

Response:

EVAR under general anesthesia was mainly performed by a vascular surgeon, and EVAR under local anesthesia was mainly performed by a radiologist. The choice between general anesthesia and local anesthesia was left to the patient. This is described in the main text (page 5, line 110 - 115)

Comment 8:
Line 136 outcomes. It would be very interesting to know data from both groups such as mortality, complications in the immediate postoperative period or the length of hospital stay.

Response:

Thank you for your comment. The immediate perioperative period was set to 30 days after the procedure. As shown in Lines 232 - 238 and Table 2, during the immediate perioperative period, there were no CVAs or deaths in either group except for one MI in the OSR group.

Comment 9:
Line 155 the authors do not have to describe what the EQ-5D is. It would be important that the authors indicate how often and when the questionnaire was passed to the patients. It is to be expected that at some moments of the follow-up there would be differences between the groups.

Response:

We agree with you and have deleted the sentences.

In 2020, when the study was in progress, a batch telephone survey was conducted to investigate the EQ-5D average for 3 years after the procedure. This is described in the main text (page 7, line 169 - 171)

Comment 10:
Table 3 is misleading in interpretation. When the 1st complication including reoperation is indicated, really in the OSR group no patient required reoperation due to the pathology. Redo the statistical analysis removing these three patients from the study.

Response:

This study aimed to compare cost-effectiveness between EVAR and OSR. Medical expenses comprised the costs for in hospital care, routine follow-up, and complication. Table 3 shows the costs associated with the complication and as mentioned in the method, complications that require readmission and incur significant costs were included. Although the complication of OSR is not a complication caused by pathology, the authors think it is right to include it because it is a complication that has affected the quality of life of patients and caused significant costs.

Comment 11:
Table 4 also leads to false interpretation. The average cost of the process is indicated. Include the total cost of complications in each of the groups.

Response:

Thank you for your comment. We included the total cost of complication in table 4.

Comment 12:
Table 5 please review the data of in-hospital cost EVAR since the cost reported is not included in the cost range

Response:

Thank you for your comments. It was a typo in the process of converting South Korean won to US dollar (USD, $). We have corrected it correctly.

Comment 13:
The same happens in table 6.

Response:

We have corrected it correctly. Thank you for your comments again.

Comment 14:
In the discussion section, the authors include data that are part of the results, for example the mean stays or the long-term survival rate. Please, review it.

Response:

We mentioned mean stay in the discussion because we wanted to emphasize the unique medical circumstance of Korea. Previous studies have shown that the lower EVAR cost compared to OSR is due to shorter hospital stays. In our study, the hospital stay of EVAR was shorter than that of OSR, but unlike other studies, the cost of EVAR was higher because of Korea's unique medical circumstance.

Our study showed survival results for patients under 70 years of age. Another study from our center that studied long-term survival involving patients over 70 years of age also wanted to emphasize that OSR is superior to EVAR.

Comment 15:
The complications that appear in the limitations must be transferred to the results, furthermore this is not a limitation, if it could be the sample size but not the appearance of these complications.

Response:

Thank you for your comment. We agree with your points. We have deleted the paragraph.

Comment 16:
Consider including in the limitations the form of inclusion of patients in each of the two branches of the study. In addition, it can also be a limitation not knowing what caused the same procedure to be performed on some occasions by a radiologist and on others by a surgeon. Another limitation of the study is the cost analysis, the authors do not individually consider the cost of staff (surgeons, radiologists, nurses)

Response:

Thank you for your comment. We agree with some points. Authors should consider having the process of dividing patients into either EVAR or OSR as a limitation. Also, one can consider the factors that led different specialists perform identical procedures a limitation. We have revised our manuscript accordingly. However, the authors are confused about the 'cost of staff". Cost of staff (procedure cost) were considered in our cost analysis. In Korea, government regulates procedure cost (including sum of all labor costs) of a surgery and therefore is standardized and is reflected in our cost analysis. (page 21, line 418 - 420)

*“Furthermore, although vascular surgeons and radiologists discussed with each other, it is possible that different specialists performed the same procedure may affect the results.”*

Comment 17:
PRISMA checklist should be added as supplemental table.

Response:

We have added the checklist as the reviewer recommended. We have added STROBE checklist because it is more suitable to our study than PRISMA checklist, which is more geared towards systematic review or meta-analysis.

**Response to Reviewer #2**

Thank you for reviewing our manuscript. Our answers to your queries are as follows.

Comment 1:

- Purpose: To evaluate quality of life and cost-effectiveness up to a 3-years FU between EVAR and OSR in patients under 70-y-o with a surgical aneurysm of abdominal aorta. The authors base their analysis on QALY which stands for “Quality Adjusted Life Year”. The QALY is commonly used in health economic evaluations as a means of quantifying the health effect of a medical intervention or a prevention program and ultimately to help payers allocate healthcare resources. It is however rather imprecise to determine the true QOL for which specific scales are proposed (but not used in the present study). I therefore recommend that the title of the manuscript would be changed accordingly: “Quality Adjusted Life Year comparison at medium term follow-up….”

Response:

Thank you for your comments. It was something we were also concerned about. I agree with your comment and changed the title. "Quality Adjusted Life Year comparison at medium term follow-up of endovascular versus open surgical repair for abdominal aortic aneurysm in young patients "

Comment 2:
- Methods: Retrospective study including 90 patients <70-y-o for whom aortic morphology would have allowed to perform TEVAR and who underwent TEVAR (37) or OSR (53) between January 2012 and October 2016.
• It is well known that studies which retrospectively screen if certain patients would have been eligible for one interventional treatment rather than for another one are flawed by many biases. Among those biases are the technological evolution of imaging, materials, and indications as well as the improvement of the individual experience and global quality of care. Those biases tend to render rather futile any comparison of patient populations treated at different time and to invalid in a more recent patient population some general results found in an historical study. For instance to compare patients who, very restrictively, benefited from TAVR between 2012 and 2016 which those who are liberally treated nowadays with TAVR updated prostheses does not make much sense. Even worse if we consider studying patients who “might have benefited from TAVR between 2012 and 2016”. To deal with such biases in the current study the authors chose to select only patients who met the nowadays aortic morphology criteria for EVAR and retrospectively reviewed all preoperative CT.

- It is important to precise who is behind the “we analyzed” preoperative CT. Was it the same observer for all CT? Were results double checked? Was a certified radiologist present among authors? Which imaging software was used?

Response:

As a mentioned in the method, all patients underwent preoperative contrast-enhanced CT. After reading this image by a professional radiologist at the hospital, one of the authors analyzed the aortic morphology on a consistent basis referring to the size mentioned. This part has been modified from the manuscript. (page 4, line 88 - 91)

*“Following the analysis of the CT images by a professional radiologist at the hospital, one of the authors analyzed whether the aortic morphology conformed to a composite list of instructions for use to include only patients with aortic morphology that was suitable for both EVAR and OSR”*

Comment 3:
- It is crucial therefore that the authors make it clear in “Methods” that not a single patient from the (historical) EVAR group did not meet the nowadays aortic morphology criteria for EVAR because this might significantly increase the risk of impaired outcomes during follow-up.
• On the other hand, to avoid including patients that might have not tolerate OSR, the authors have excluded 14 patients for EVAR who would not have tolerate general anaesthesia. However the EVAR group did include 18 procedures (50%) performed under local anaesthesia. The authors must therefore precise the criteria of choice for local versus general anaesthesia in the EVAR group and how they can retrospectively be sure that patients who underwent EVAR under LA could have tolerate GA as well?

Response:

The authors agree with you and think that this part needs more explanation. Even when EVAR was always performed under local anesthesia, we prepared for general anesthesia to be possible just in case. Risk factors were assessed and necessary tests were performed. When performed under local anesthesia, the patients had the advantage of reducing the risk of general anesthesia. However, patients had to lie on a hard bed during long procedures and became anxious. After fully explaining these factors, it was decided to choose between local anesthesia and general anesthesia. This part has been added from the manuscript. (page 5, line 103 - 110)

*“Even when EVAR was always performed under LA, we prepared for GA to be possible just in case*

*The choice between EVAR and OSR was based on patient preference. After the preoperative evaluation, the patients made a decision after consulting the doctor in charge (vascular surgeon and radiologist) about the treatment options. When performed under LA, the patients had the advantage of reducing the risk of GA. However, patients had to lie on a firm bed during the long procedures and became anxious. The patients carefully considered the strengths and weaknesses of each treatment and chose accordingly.”*

Comment 4:
- Results: Even in case of a favourable anatomy, a lesser invasive treatment tend to be proposed in frail or disable patients. Patients with a past history of cancer are also good candidate for a lesser invasive treatment. The authors present a baseline comparison of demographics (Table 1) which is in line with this general figure. Due to the low number of patients and to assess a better comparison between both groups (taking into account that this is not a matched study), I strongly recommend that the authors present in Table 1 the Charlson Comorbidity Index for EAVR and OSR patients.

Response:

Thank you for your comment. We have added the Charlson Comorbidity Index in Table 1 as the reviewer recommended.

Comment 5:
• A higher rate of complications is expected with EVAR during follow-up and patients must stay therefore under imaging surveillance for a life time which impacts QOL. Some large recent randomized trials share those unequivocal conclusions, however a lower global survival with EVAR in the long term is not unanimously found (please do actualise bibliography, for instance we expect to find DOI: 10.1056/NEJMoa1715955). I strongly recommend to present actuarial survival curves (freedom from death of any cause, freedom of death from aortic cause, and freedom of reintervention) in a Figure 1.

Response:

We agree with your opinion. A survival curves (freedom from all-cause death, freedom from aneurysm-related death, freedom from complication (requiring readmission) were added as figure 2 to the follow-up result. There was no statistically significant difference in all-cause death and aneurysm-related death between the two groups, but there was a significant difference in complication (requiring readmission) free survival.

Comment 6:
• HRQOL (Health Related Quality of Life) is usually assessed by a score on a HRQOL-14 items scale. Since such a scale has not been used, I recommend the authors to strictly stay on the notions of QALY which can be assimilated to the crude survival in complete heath for each group and cost per QALY which are relevant in this study up to a medium term FU.

Response:

Thank you for your comment. It seems that we were using the word HRQOL confusingly. The word HRQOL was used only in the meaning of health-related QOL, not in the meaning of scale. Parts that can be misunderstood as scale have been modified in expression. (page 3, lines 73 – 76; page 7, lines 150 – 153; page 17, lines 334 - 339)

“This study compared quality-adjusted life years (QALYs) and medical expenses and reported complication rates, including reinterventions associated with increased cost, in young patients (<70 years) with suitable aortic anatomy for EVAR and OSR.”

“The secondary outcomes were readmission-requiring complication rates including reintervention that brought cost increase and QALY change, all-cause death, and aneurysm-related death in the perioperative period (30 days) and during follow-up.”

“The main reason for the EVAR long-term outcome being lower than OSR long-term outcome was the reintervention rate. The high rate of reintervention is related with the high cost and QALY. Young populations who have a longer life expectancy than old populations are more exposed to chances for reintervention, leading to a high cost and low QALY. These young patients with AAA who fit both OSR and EVAR were our subject of investigation into the reintervention rate, medical expenses, and QALY.”

Comment 7:
• Finally minor English revisions are required, please check carefully manuscript (for instance: “Heria repair” in Table 4; “Fallow-up cost” in Table 6; “follow-ups” in Cost analysis…..)

Response:

Thank you for your careful comment. English correction was performed once more to correct all typos.

Thank you again for reviewing our manuscript in detail and providing helpful comments. We hope that our responses and the corresponding revisions are satisfactory.
